# The effect of 60 days of 6° head-down-tilt bed rest on circulating adropin, irisin, retinol binding protein-4 (RBP4) and individual metabolic responses in young, healthy males

**DOI:** 10.3389/fphys.2024.1435448

**Published:** 2024-09-10

**Authors:** Kiera Ward, Edwin Mulder, Petra Frings-Meuthen, Donal J. O’Gorman, Diane Cooper

**Affiliations:** ^1^ Faculty of Science and Health, Technological University of the Shannon, Athlone Campus, Athlone, Ireland; ^2^ Department of Muscle and Bone Metabolism, Institute of Aerospace Medicine, German Aerospace Center (DLR), Cologne, Germany; ^3^ 3U Diabetes Partnership, School of Health and Human Performance, Dublin City University, Dublin, Ireland; ^4^ National Institute for Cellular Biotechnology, Dublin City University, Dublin, Ireland; ^5^ EduFIT Limited, Laois, Ireland

**Keywords:** adropin, irisin, RBP4, liver, bed rest, biomarkers and individual metabolic responses

## Abstract

**Background:**

Alterations in the circulating concentrations and target-tissue action of organokines underpin the development of insulin resistance in microgravity and gravity deprivation. The purpose of this study was to examine changes in circulating adropin, irisin, retinol binding protein-4 (RBP4), and the metabolic response of healthy young males following 60 days of 6° head-down-tilt (HDT) bed rest, with and without reactive jump training (RJT), to explore links with whole-body and tissue-specific insulin sensitivity. To our knowledge, this is the first time that adropin, irisin, and RBP4 have been studied in HDT bed rest.

**Methods:**

A total of 23 male subjects (29 ± 6 years, 181 ± 6 cm, 77 ± 7 kg) were exposed to 60 days of 6° HDT bed rest and randomized to a control (CTRL, n = 11) or a RJT (JUMP, n = 12) group (48 sessions with ≤4 min total training time per session). Circulating adropin, irisin, and RBP4 were quantified in fasting serum before and after HDT bed rest. A subanalysis was performed *a posteriori* to investigate individual metabolic responses post-HDT bed rest based on subjects that showed an increase or decrease in whole-body insulin sensitivity (Matsuda index).

**Results:**

There were significant main effects of time, but not group, for decreases in adropin, irisin, Matsuda index, and liver insulin sensitivity following HDT bed rest (*p* < 0.05), whereas RBP4 did not change. The subanalysis identified that in a subgroup with decreased whole-body insulin sensitivity (n = 17), RBP4 increased significantly, whereas adropin, irisin, and liver insulin sensitivity were all decreased significantly following HDT bed rest. Conversely, in a subgroup with increased whole-body insulin sensitivity (n = 6), liver insulin sensitivity increased significantly after HDT bed rest, whereas adropin, irisin, and RBP4 did not change.

**Conclusion:**

Investigating individual metabolic responses has provided insights into changes in circulating adropin, irisin, RBP4, in relation to insulin sensitivity following HDT bed rest. We conclude that adropin, irisin, and RBP4 are candidate biomarkers for providing insights into whole-body and tissue-specific insulin sensitivity to track changes in physiological responsiveness to a gravity deprivation intervention in a lean male cohort.

## 1 Introduction

The amount and behavior of skeletal muscle, adipose tissue, and the liver, has a substantial impact on metabolism and physiology, including the production and secretion of myokines, adipokines and hepatokines, respectively (de Oliveira dos Santos et al., 2021). These pleiotropic molecules, collectively known as organokines, regulate inflammation, energy, and metabolic homeostasis by exerting various autocrine, paracrine, and endocrine actions. The atrophic conditions of microgravity (spaceflight) and gravity deprivation (physical inactivity, sedentary behaviour, immobilization and the limited mobility of ageing), even in energy-balanced conditions, can dramatically affect metabolism and physiology, causing muscle deconditioning, bone loss, and metabolic dysregulation, particularly insulin resistance (Vernikos, 2017; Vernikos, 2021; Demontis et al., 2017; Tanaka et al., 2017). The atrophic effect differs only in the rate at which it is induced, determined by the lifestyle use of the gravity vector. Investigation of circulating organokines and their roles in inter-organ crosstalk, has the potential to identify and understand the etiology of insulin resistance under these conditions (Priest and Tontonoz, 2019).

**FIGURE 1:**
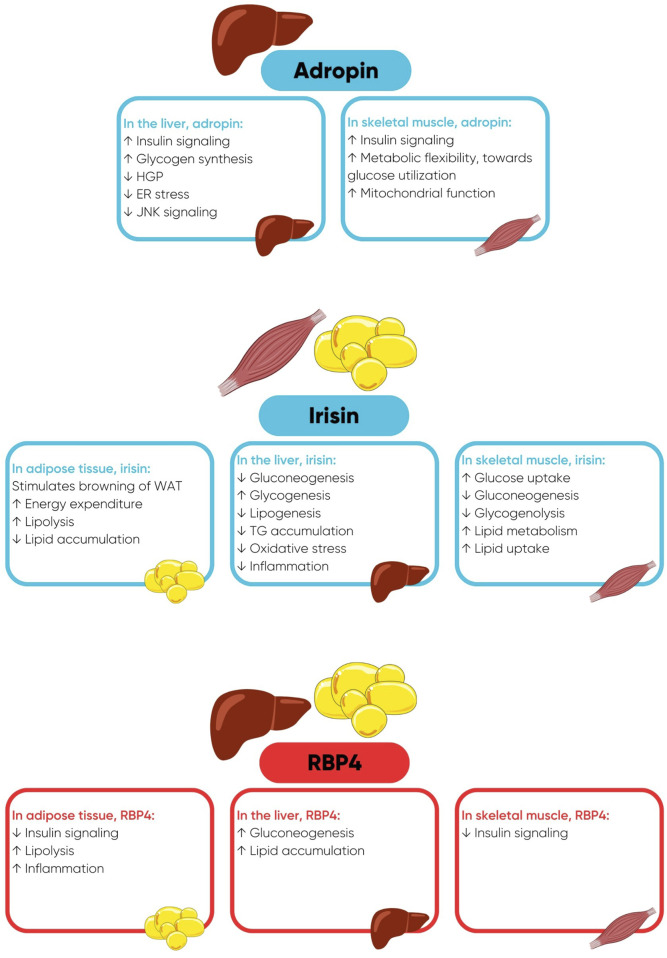
Favourable metabolic actions of adropin and irisin and deleterious metabolic actions of retinol binding protein-4 (RBP4), when concentrations of RBP4 are elevated. Abbreviations: ER, endoplasmic reticulum; HGP, hepatic glucose production; JNK, c-Jun N-terminal Kinase; TG, triglycerides; WAT, white adipose tissue.

Adropin has a regulatory role in substrate oxidation and sensitizes insulin-stimulated intracellular pathways in the liver and skeletal muscle (Figure 1) (Kolben et al., 2020; Gao et al., 2019; Gao et al., 2014; Gao et al., 2015). Adropin also has beneficial effects on the cardiovascular system by reducing arterial stiffness and improving endothelial function (Fujie et al., 2017; Fujie et al., 2015; Zhang et al., 2017; Kolben et al., 2020). Irisin is a polypeptide hormone that is mainly secreted from skeletal muscle in response to physical activity (Boström et al., 2012; Arhire et al., 2019). Adipose tissue is also a source of irisin, however, in humans, the expression of fibronectin type III domain-containing protein 5 (FNDC5), the precursor of irisin, is 200 times lower in adipose tissue compared with skeletal muscle (Moreno-Navarrete et al., 2013; Perakakis et al., 2017). Irisin has a multitude of beneficial functions in metabolic organs including adipose tissue, skeletal muscle, and the liver (Figure 1), maintaining normoglycemia and normolipidemia and enhancing insulin sensitivity (Perakakis et al., 2017; Arhire et al., 2019; Park S. E. et al., 2015; Liu et al., 2015). Retinol binding protein-4 (RBP4) is secreted by the liver, and to a lesser extent by adipose tissue, and is the predominant transport protein for retinol/vitamin A in circulation. In comparison to adropin and irisin, RBP4 is involved in the pathogenesis of insulin resistance by exerting numerous deleterious metabolic actions in adipose tissue, skeletal muscle, and the liver (Figure 1) (Yang et al., 2005; Ost et al., 2007; Norseen et al., 2012; Lee et al., 2016).

Accordingly, the balance between the secretions of these two different types of organokines will either promote or deter metabolic dysfunction. Physical inactivity and high levels of sedentary time impair the secretion of health-enhancing organokines and promote the secretion of organokines that contribute to the development of metabolic diseases. Contrastingly, exercise training enhances the secretion of organokines that induce favorable changes in local and systemic metabolism (Leal et al., 2018). Hence, investigation of these circulating organokines and thus the behaviour of key metabolic organs may improve our understanding of the complex relationship of these metabolic networks and inter-organ communication and provide insights into individual metabolic responses in insulin sensitivity and metabolic deregulation following HDT bed rest, an extreme model of physical inactivity and sedentary behaviour. To our knowledge, changes in circulating adropin, irisin, and RBP4 have not been measured previously in response to HDT bed rest. Therefore, the purpose of this study was to examine changes in circulating adropin, irisin, RBP4, and the metabolic response of healthy young males following 60 days of 6° HDT bed rest, with and without reactive jump training (RJT), to explore links with whole-body and tissue-specific insulin sensitivity.

## 2 Materials and methods

### 2.1 General study information

This research was performed as part of the “Reactive jumps in a sledge jump system as a countermeasure during long-term bed rest” (RSL) study, which was conducted at the German Aerospace Center (DLR): envihab facility and funded by the European Space Agency, in 2015/2016. Information on subject recruitment, experimental conditions, diet, exercise countermeasure, training protocol, and collection of baseline core data including body composition, body weight, and peak oxygen uptake ( $\dot{V}$ O_2peak_) specific to this parallel-design randomized controlled training study have been reported previously (Kramer et al., 2017a; Kramer et al., 2017b; Ward et al., 2020). Additionally, information on the oral glucose tolerance test, biochemical analysis and assays (glucose, insulin, lipid profile) and calculation of indexes of insulin sensitivity (Matsuda index and liver insulin sensitivity) have been published previously (Ward et al., 2020).

Briefly, the study consisted of a 15-day baseline data collection phase (BDC-15 to BDC-1), 60 days of 6° head-down-tilt bed rest (HDT1 to HDT60) and a 15-day recovery phase (R+0 to R+14). For the duration of the HDT bed rest phase, subjects remained at the 6° HDT angle for 24 h/day and were randomly assigned to a control group (CTRL) or an intervention group involving reactive jump training (JUMP). Basic inclusion criteria were specified as follows: male, aged between 20 and 45 years, body mass index (BMI) between 20 and 26 kg/m^2^, non-smoking, taking no medication, non-competitive athletes, and no history of bone fractures. A total of 24 male volunteers were enrolled for this study, however, one subject was withdrawn during BDC due to medical reasons unrelated to the study and could not be replaced.

On HDT1, stratified pairs of subjects (similar age, height, and weight) were randomly assigned to either the CTRL group (n = 11, age 28 ± 6 years, BMI 23.3 ± 2 kg/m^2^) or the JUMP group (n = 12, age 30 ± 7 years, BMI 23.8 ± 2 kg/m^2^) using a dice roll. Two subjects (one CTRL, one JUMP) were re-ambulated on HDT49 and HDT50, respectively, due to medical reasons but completed the scheduled measurements relevant to this study (i.e., post-HDT bed rest fasted blood draw). The JUMP group performed RJT 6 days per week (48 sessions in total). On average, each session consisted of 48 countermovement jumps and 30 hops, performed with maximal effort at a load equal to 80%–90% body weight in a custom-designed horizontal sledge jump system (≤4 min total exercise time). During each stage of the study, subjects received a strictly controlled and individualized diet, which was tailored to maintain energy balance. The study protocols were approved by the Ethics Committee of the North Rhine Medical Association (Ärztekammer Nordrhein) in Düsseldorf, Germany, and the Federal Office for Radiation Protection (Bundesamt für Strahlenschutz). All subjects gave written informed consent before the start of the study in accordance with the Declaration of Helsinki. The RSL study was registered retrospectively with the German Clinical Trial Registry (#DRKS00012946, 18th September 2017).

### 2.2 Quantification of circulating biomarkers

Using fasting serum samples collected in the morning of BDC-5 (pre) and HDT59 (post), the concentrations of adropin, irisin, and RBP4 were assayed in duplicate according to the manufacturer’s instructions. Specifically, adropin was quantified with the Novus Biologicals Human Adropin ELISA (Cat# NBP2-66433; intra-assay CV: 13.61%), irisin was quantified using the BioVendor Human Irisin ELISA (Cat# RAG018R; intra-assay CV: 8.57%) and RBP4 was quantified using the BioVendor Human RBP4 ELISA (Cat# RAG005R; intra-assay CV: 8.93%).

### 2.3 Plasma volume correction

The final concentrations of adropin, irisin, and RBP4 following HDT bed rest were corrected for changes in hemoconcentration, as the 6° HDT angle is known to induce a fluid shift and consequently a loss of plasma volume (PV) (Pandiarajan and Hargens, 2020). The change in PV (Δ%PV) was calculated as follows: Δ%PV = 100*[(Hb_pre_/Hb_post_)*(100–Htc_post_)/(100–Htc_pre_)–1], where hemoglobin (Hb) is provided in g/dL and hematocrit (Hct) is expressed as a percentage (%). To correct measured parameters for changes in PV, the following calculation was applied: [parameter]c = [parameter]u * [1 + ΔPV(%)/100], where the c and u indices represent the corrected and uncorrected final concentrations, respectively (Dill and Costill, 1974; Alis et al., 2016).

### 2.4 Statistical analysis

Data is presented as mean ± standard deviation (SD). Normality of distribution was evaluated using either the Shapiro-Wilk test or by inspecting residuals on a Q-Q Plot, and data violating the assumption of normality was transformed. Differences in baseline characteristics between the two experimental groups (CTRL, JUMP) were assessed using independent samples t-tests or the Welch t-test. Physical and metabolic changes in response to HDT bed rest were analysed using a mixed between-within factorial analysis of variance (or mixed ANOVA) using time as the within-group factor (pre, post) and group as the between-group factor (CTRL, JUMP). Statistical analysis was performed using SPSS 26.0 (IBM Corp., Armonk, NY, United States) considering a two-sided 0.05 significance level.

### 2.5 Subgroup statistical analysis

In an attempt to further understand the different individual metabolic responses post-HDT bed rest, the subject data from the two groups (CTRL, JUMP) was pooled and divided into two subgroups based on individuals who displayed decreased (↓ Matsuda, n = 17, CTRL n = 7, JUMP n = 10) or increased (↑ Matsuda, n = 6, CTRL n = 4 and JUMP n = 2) insulin sensitivity post-HDT bed rest. Normality of distribution was evaluated specific to the statistical procedure being conducted. Differences in baseline characteristics between the two subgroups were examined using independent samples t-tests or the Welch t-test. Paired sample t-tests were used to examine the significance of pre-to post-HDT bed rest changes in metabolic responses in both subgroups. Alpha adjustments were not made due to consistent use of individual testing, which permits the testing of individual null hypotheses that do not compromise joint null hypotheses (Rubin, 2021).

## 3 Results

### 3.1 Biomarker and metabolic responses

**FIGURE 2:**
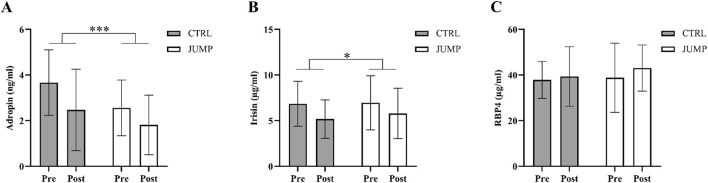
The effect of 60 days HDT bed rest on circulating adropin **(A)**, irisin **(B)**, and RBP4 **(C)** measured on BDC-5 (pre) and HDT59 (post). Data are presented as mean ± SD. The results for adropin, irisin, and RBP4 (figure) include n = 11 in CTRL and n = 12 JUMP. Biomarker concentrations were corrected for changes in hemoconcentration post-HDT bed rest. Abbreviations: CTRL, control group; JUMP, jumping countermeasure group. A significant main effect of time indicated as *p ≤ 0.05 or ***p ≤ 0.001.

The baseline descriptive characteristics of subjects in the CTRL and JUMP groups are displayed in Table 1. Changes in circulating adropin, irisin, and RBP4 are displayed in Figure 2. As mentioned above, biomarker concentrations were corrected for changes in hemoconcentration post-HDT bed rest (mean ΔPV% CTRL 0.88%, JUMP -2.13%). There were no significant between-group differences in circulating adropin, irisin, and RBP4 at baseline. There was a significant main effect of time, but not group, for the decreases in irisin and adropin post-HDT bed rest. RBP4 did not change.

**TABLE 1:** Baseline descriptive characteristics of the subjects in the RSL bed rest study.

	CTRL (n = 11)	JUMP (n = 12)
Age (years)	28 ± 6	30 ± 7
Height (cm)	181 ± 5	181 ± 7
BW (kg)	76.10 ± 8.06	77.85 ± 6.55
BMI (kg/m^2^)	23.33 ± 2.03	23.75 ± 1.80

	CTRL (n = 10)	JUMP (n = 10)
$\dot{V}$ O_2peak_ (L/min)	3.85 ± 0.68	3.32 ± 0.76

Data are presented as mean ± standard deviation (SD). Abbreviations: CTRL, control group; JUMP, jumping countermeasure group; N, number; BW, body weight; BMI, body mass index; $\dot{V}$ O_2peak_, peak aerobic capacity. $\dot{V}$ O_2peak_ measurements were available in 20 subjects only due to the absence of post-HDT bed rest data and failure to meet the test criteria.

Changes in metabolic responses (glucose, insulin, and lipids) and indexes of insulin sensitivity (Matsuda index and liver insulin sensitivity) in the CTRL and JUMP group have been reported previously (Ward et al., 2020) and are provided in Supplementary Material. Briefly, there were no significant between-group differences in metabolic responses at baseline. There was a significant effect of time, but not group, for increases in glucose_60_, glucose_120_, AUCG_120_, AUCI_120_, triglycerides and LDL-cholesterol, and decreases in HDL-cholesterol and the Matsuda index. A significant effect of time and group was found for insulin_0_, which increased significantly following HDT bed rest and was significantly higher overall in the JUMP group. Liver insulin sensitivity decreased significantly post-HDT bed rest and was significantly lower, overall, in the JUMP group.

### 3.2 Subanalysis exploring the individual metabolic responses

#### 3.2.1 Changes in metabolic responses

Baseline descriptive characteristics, when subjects were divided into two subgroups based on a decrease or an increase in insulin sensitivity post-HDT bed rest, based on the Matsuda index, are displayed in Table 2. Changes in metabolic responses and indexes of insulin sensitivity in the subgroups with decreased and increased insulin sensitivity following HDT bed rest were published previously and are presented in Supplementary Material.

**TABLE 2:** Baseline descriptive characteristics when subjects were divided into two subgroups based on a decrease or an increase in insulin sensitivity post-HDT bed rest.

	Decreased insulin sensitivity subgroup (n = 17)	Increased insulin sensitivity subgroup (n = 6)
Age (years)	29 ± 6	30 ± 7
Height (cm)	181 ± 6	180 ± 6
BW (kg)	76.56 ± 7.51	78.30 ± 6.68
BMI (kg/m^2^)	23.37 ± 1.97	24.06 ± 1.67

	Decreased Insulin Sensitivity Subgroup (n = 15)	Increased Insulin Sensitivity Subgroup (n = 5)
$\dot{V}$ O_2peak_ (L/min)	3.61 ± 0.78	3.51 ± 0.75

Data are presented as mean ± SD. Abbreviations: N, number; BW, body weight; BMI, body mass index; $\dot{V}$ O_2peak_, peak aerobic capacity. $\dot{V}$ O_2peak_ measurements were available in 20 subjects only due to the absence of post-HDT bed rest data and failure to meet the test criteria.

To summarise, the analysis of between-subgroup differences in glucose, insulin, and lipid responses at baseline found a statistically significant difference in insulin_30_ only, with higher mean values in subjects with increased insulin sensitivity following HDT bed rest (*p* = 0.050). In the subgroup of individuals who became less-sensitive after HDT bed rest, the pre-to post-increases in glucose_0_, glucose_60_, glucose_90_, glucose_120_, AUCG_30_, AUCG_120_, insulin_0_, AUCI_120_, triglycerides and LDL-cholesterol and the pre-to post-decrease in HDL-cholesterol, were statistically significant. In the subgroup of individuals who became more insulin-sensitive following HDT bed rest, the pre-to post-decreases in AUCG_30_, insulin_30_, AUCI_30_ and HDL-cholesterol were statistically significant. Examination of between-group differences in indexes of insulin sensitivity at baseline identified a statistically significant difference in liver insulin sensitivity only, with higher mean values in subjects who became less insulin sensitive following HDT bed rest (*p* = 0.048). In the subgroup of individuals who became less insulin-sensitive post-HDT bed rest, the pre-to post-decrease in liver insulin sensitivity was statistically significant. In the subgroup of individuals who became more insulin-sensitive following HDT bed rest, the pre-to post-increase in liver insulin sensitivity was statistically significant.

#### 3.2.2 Changes in circulating biomarkers

**FIGURE 3:**
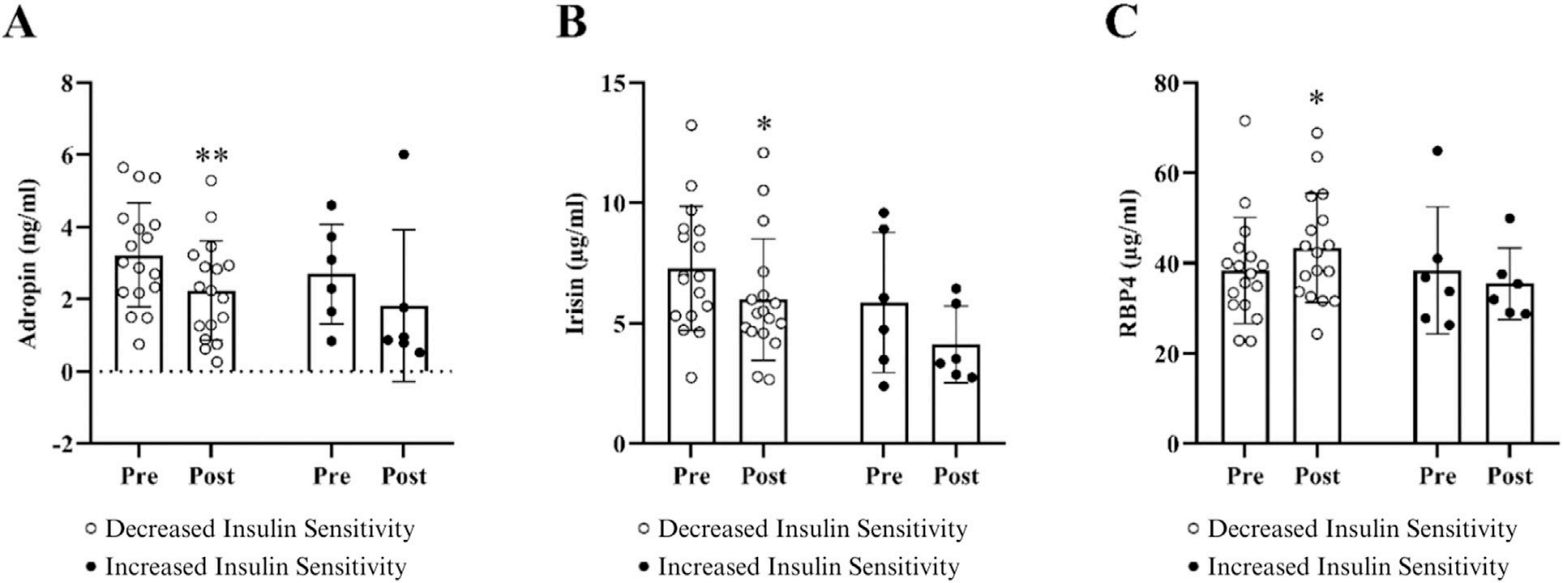
Changes in circulating adropin **(A)**, irisin **(B)**, and RBP4 **(C)** when subjects were divided into two subgroups based on a decrease or an increase in insulin sensitivity post-HDT bed rest. Data are presented as mean ± SD. Metabolic characteristics were measured on BDC-5 and HDT59. Pre-to post-HDT bed rest changes in adropin, irisin, and RBP4 are shown in figure, respectively. Biomarker concentrations were corrected for changes in hemoconcentration post HDT-bed rest. Abbreviations: RBP4, retinol binding protein 4. The results for these circulating biomarkers include n = 17 in the decreased insulin sensitivity subgroup and n = 6 in the increased insulin sensitivity subgroup. *p ≤ 0.05 or **p ≤ 0.01.

Changes in circulating adropin, irisin, and RBP4 in the subgroups with decreased and increased insulin sensitivity following HDT bed rest are displayed in Figure 3. No significant between-subgroup differences in circulating adropin, irisin, and RBP4 were noticeable at baseline. In the subgroup of individuals who became less insulin-sensitive after HDT bed rest, the pre-to post-increase in RBP4 and pre-to post-decreases in adropin and irisin were statistically significant. Contrastingly, in the subgroup of individuals who became more insulin-sensitive following HDT bed rest, adropin, irisin, and RBP4 did not change following HDT bed rest.

## 4 Discussion

The findings of the present study show that 60 days of 6° HDT bed rest significantly reduced circulating concentrations of adropin and irisin, concomitant to impaired insulin action and decreased peripheral glucose uptake, in healthy lean males, irrespective of whether subjects performed RJT or not. Conversely, circulating concentrations of RBP4 did not change, despite evidence that factors which influence this biomarkers secretion changed in response to HDT bed rest (e.g., muscle atrophy, $\dot{V}$ O_2peak_, and insulin sensitivity) (Ward et al., 2020). To our knowledge, this is the first time that RBP4, irisin, and adropin have been studied under conditions of HDT bed rest.

Adropin is a circulating hepatokine, which modulates metabolic homeostasis by exerting systemic effects on insulin sensitivity (Mushala and Scott, 2021). Adropin acts on the liver and skeletal muscle and enhances insulin signalling and metabolic flexibility and reduces hepatic glucose production (HGP), endoplasmic reticulum stress, and c-Jun N-terminal Kinase (JNK) activity (Gao et al., 2019; Gao et al., 2014; Gao et al., 2015). Liver dysfunction decreases adropin expression and triggers insulin resistance (Mushala and Scott, 2021). Accordingly, lower circulating concentrations of adropin have been reported in humans with obesity, insulin resistance, type 2 diabetes mellitus (T2DM), and hepatosteatosis (Butler et al., 2012; Zang et al., 2018; Kutlu et al., 2019). The reduction in circulating adropin may have contributed to impaired insulin signalling, leading to reduced peripheral glucose uptake and clearance of triglycerides from circulation, which was compounded with the reduction of skeletal muscle mass following prolonged inactivity and high levels of sedentary time (Ward et al., 2020; Kramer et al., 2017b), a key target organ mediating the favorable metabolic effects of adropin (Gao et al., 2014). While we did not measure changes in metabolic flexibility in this study, previous research has reported that HDT bed rest, induces glucose intolerance that is preceded by a metabolically inflexible state (Rudwill et al., 2018; Le Roux et al., 2021), which could be impacted by lower concentrations of adropin.

Irisin is secreted primarily from skeletal muscle in response to exercise but is also secreted in small amounts by adipose tissue. Irisin maintains glucose and lipid metabolism in peripheral tissues, contributing to improvements in insulin sensitivity, reduced endoplasmic reticulum stress, and β-islet cell survival and function (Huh et al., 2014; Liu et al., 2015; Park M. J. et al., 2015; Perakakis et al., 2017; Arhire et al., 2019). Circulating irisin concentrations decrease progressively with the worsening of glucose tolerance, are reduced in T2DM and are associated with compromised expression and secretion of irisin from skeletal muscle (Choi et al., 2013; Liu et al., 2013; Assyov et al., 2016; Shoukry et al., 2016). It is possible that the pronounced reduction in lean mass (Ward et al., 2020; Kramer et al., 2017b) and contractile activity may have blunted the secretion of irisin in response to HDT bed rest, leading to reduced insulin-induced PI3K/Akt signalling and glucose disposal. Furthermore, lower concentrations of irisin as a result of extreme inactivity and sedentarism caused by HDT bed rest may have reduced lipid metabolism and lipid uptake in skeletal muscle and elevated lipogenesis in the liver, thereby contributing to hyperlipidemia post-HDT bed rest.

RBP4 is a member of the lipocalin family of transport proteins and is produced mainly by the liver, but also by adipose tissue (Bergmann and Sypniewska, 2013). RBP4 contributes to the development of insulin resistance by impairing insulin signalling in skeletal muscle and adipose tissue, upregulating lipolysis and inflammation in adipose tissue and increasing gluconeogenesis and lipid accumulation in the liver (Yang et al., 2005; Ost et al., 2007; Norseen et al., 2012; Lee et al., 2016). Higher circulating levels of RBP4 have been reported in visceral obesity, insulin resistance, prediabetes, T2DM, non-alcoholic fatty liver disease (NAFLD), and the metabolic syndrome (Cho et al., 2006; Jia et al., 2007; El-Mesallamy et al., 2013; Chen et al., 2017; Wessel et al., 2019). Fasting glucose is an independent determinant of circulating RBP4 (Cho et al., 2006). We documented no significant changes in circulating RBP4 or fasting glucose, irrespective of CTRL or JUMP group allocation, after HDT bed rest. This may reflect unchanged hepatic glucose production, as RBP4 has been shown to increase the expression of phosphoenolpyruvate carboxykinase (PEPCK), a key gluconeogenic enzyme in the liver (Yang et al., 2005).

A plethora of research has reported that different modalities of exercise training, including aerobic, resistance, and high-intensity interval training, as well as concurrent training, can improve body composition, aerobic capacity, muscle strength, intrahepatic lipid content and insulin sensitivity in healthy and clinical populations (Graham et al., 2006; Lim et al., 2008; Kim et al., 2016; Zhang et al., 2017; Murawska-Cialowicz et al., 2020). These exercise-induced changes are associated with favorable changes in organokines, which interplay through inter-organ crosstalk and induce physiological adaptations to mediate the beneficial effects of exercise. Contrastingly, there is also literature showing that circulating organokines do not change in response to different types of exercise, despite positive changes in the physical and metabolic characteristics of individuals, with and without metabolic dysfunction (Choi et al., 2009; Hecksteden et al., 2013; Croymans et al., 2013). The concentration and effect of circulating biomarkers may differ in response to exercise intensity, duration, and frequency (Gonzalez-Gil and Elizondo-Montemayor, 2020). In the present study, RJT, a form of high-intensity interval training encompassing plyometric movements with high rates of force development, elicited protective effects on muscle mass and function, including attenuating the loss of whole-body lean mass, leg lean mass, $\dot{V}$ O_2peak_, and muscle strength, as published previously (Kramer et al., 2017a; Kramer et al., 2017b; Ward et al., 2020). However, in the present work, the exercise regime was not sufficient to induce favorable changes in circulating adropin, irisin, and RBP4 or insulin sensitivity following 60 days of HDT bed rest. It is possible that the low volume of training (6 days per week, or 48 sessions in total with 48 jumps and 30 hops per session, equivalent to ≤4 min total training time per session) could not prevent the dysregulation of glucose and lipid metabolism following HDT bed rest. The primary stimulus in the current study was physical inactivity and sedentarism, as a result of strict exposure to HDT bed rest, and the exercise countermeasure was designed solely to prevent muscle and bone loss. Additionally, the divergent responses of biomarkers to the intervention as detailed above hinted towards individual metabolic responses to combined physical inactivity and sedentarism, which needed to be examined further. Through investigating individual metabolic responses in the present study, we were able to formulate and profile the physical and metabolic changes that occurred in subgroups of individuals that showed 1) decreased whole-body insulin sensitivity following HDT bed rest, or 2) increased whole-body insulin sensitivity following HDT bed rest.

The key findings in the subgroup with decreased insulin sensitivity after HDT bed rest show that, in addition to muscle atrophy and reduced cardiorespiratory fitness, there were marked impairments in insulin action and peripheral glucose uptake in the subgroup with decreased insulin sensitivity, as published previously (Ward et al., 2020). In brief, there was a significant increase in fasting insulin, glucose, and triglycerides and a significant decrease in HDL-cholesterol, as well as higher glycaemia in the last hour of the OGTT. The increase in circulating triglycerides and decrease in HDL-cholesterol may represent resistance of the ability of insulin to suppress VLDL production in individuals who became less insulin-sensitive post-HDT bed rest. In addition to a significant reduction in the Matsuda index, there was a significant decrease in liver insulin sensitivity in this subgroup following HDT bed rest. Circulating concentrations of RBP4 increased significantly and circulating concentrations of adropin and irisin decreased significantly in the subgroup with decreased insulin sensitivity after HDT bed rest. The increase in RBP4 and decreases in adropin and irisin were expected given the observed decline in insulin sensitivity in this subgroup post-HDT bed rest. In line with these findings, our previous publication reports that circulating concentrations of fetuin-A also increased significantly in the subgroup with decreased insulin sensitivity following HDT bed rest (Ward et al., 2020). Thus, this panel of four biomarkers (adropin, irisin, RBP4, and fetuin-A) may be useful to track changes in systemic and tissue-specific metabolic health in response to HDT bed rest in a lean population.

The main findings in the subgroup with increased insulin sensitivity following HDT bed rest provide evidence that circulating concentrations of RBP4, adropin, and irisin did not change significantly. Results from our previous publication indicate that circulating concentrations of fetuin-A did not change significantly in the subgroup with improved insulin sensitivity following HDT bed rest (Ward et al., 2020). These observations are noteworthy because these organokines all changed unfavorably in response to a decline in insulin sensitivity after HDT bed rest. Fetuin-A and RBP4 impair insulin signaling, whereas adropin and irisin improve insulin signalling, which suggests that it is possible that the improvement in insulin sensitivity in this subgroup may be related to the maintenance of normal insulin signalling in peripheral tissues. Additionally, there was a substantial improvement in liver insulin sensitivity in the subgroup with increased insulin sensitivity post-HDT bed rest, indicated by significant decreases in insulin concentrations 30 min after the glucose load, 30-min AUC values for glucose and insulin, and a significant increase in Matsuda index and liver insulin sensitivity. Therefore, in agreement with our first publication (Ward et al., 2020), we propose that the improvement in whole-body insulin sensitivity in this subgroup may be an indirect effect of liver metabolism. We acknowledge that the sample size in the subgroup with increased insulin sensitivity is small (n = 6, CTRL n = 4, JUMP n = 2). However, the observation that liver insulin sensitivity improved in this subgroup following extreme inactivity and sedentarism is interesting and highlights the importance of examining individual responses to any intervention.

The findings presented above highlight that, while it is not normally a prime consideration during study design, the analysis of individual physiological and metabolic responses has relevant and important implications in many areas. For space agencies, the examination of individual responses may help to individually tailor countermeasure strategies to preserve metabolic physiology despite microgravity exposure. For public health agencies, the examination of individual responses has practical implications for health management and personalised exercise recommendations for physically inactive, sedentary, and ageing populations, particularly those with sarcopenia, osteosarcopenia, sarcopenic obesity, or other metabolic conditions (e.g., prediabetes or T2DM), who may or may not be immobilized due to hospitalization, illness, or injury. Furthermore, the quantification of circulating organokines is a minimally invasive process of exploring and understanding the complexities of insulin resistance, which is an eminent consequence of gravity deprivation. Measuring a panel of biomarkers of insulin sensitivity and insulin resistance has the potential to provide new comprehensive assessments of alterations in biological processes and changes in underlying mechanisms of glucose regulation, permit early detection and screening of insulin resistance thereby allowing for early intervention and therapeutic monitoring, and contribute to personalised medicine and optimization of exercise prescriptions for space agencies and public health agencies. By quantifying circulating adropin, irisin, and RBP4 and performing subgroup analysis, it is postulated that the maintenance of insulin signalling in peripheral tissues, in addition to the improvement in liver insulin sensitivity, may have contributed to an improvement in whole-body insulin sensitivity in the subgroup with increased whole-body insulin sensitivity post-HDT bed rest, whereas the deterioration of whole-body insulin sensitivity following HDT bed rest in the opposing group may be a consequence of impairments in the liver and skeletal muscle. These results provide a foundation for future research to progressively elucidate the physiological functions of adropin, irisin and RBP4 and highlight that the liver remains an important organ to investigate under the conditions of physical inactivity and sedentarism. As no direct measurements of liver fat have been performed in humans for ethical reasons, it is important that future research continues to investigate the effect of prolonged inactivity and high levels of sedentary time on additional circulating hepatokines to determine how changes in liver metabolism affect other peripheral organs and systemic metabolic homeostasis.

Despite the highly-controlled nature of this HDT bed rest study, several limitations have to be disclosed. Firstly, we acknowledge that the analysis of individual metabolic responses post-HDT bed rest was conducted *a posteriori* and therefore, a power analysis was not conducted. Secondly, we acknowledge that the quantification of individual response heterogeneity to any intervention is dependent on sufficient sample size, inclusion of an ambulatory control group and appropriate statistical analyses (Fernandez-Gonzalo et al., 2021; Böcker et al., 2022), and therefore must be considered during the design phase of future studies with an aim of investigating individual variability. Thirdly, we recognize potential volunteer bias and note that the results of this study are delimited to healthy lean men. Similar investigations need to be extended to other populations (e.g., healthy lean women and metabolically unhealthy and/or ageing men and women, from diverse backgrounds) to provide additional insights into changes in metabolic physiology and circulating biomarkers in response to physical inactivity and sedentarism.

## 5 Conclusion

To conclude, we report that 60 days of 6° HDT bed rest significantly reduced circulating adropin and irisin, concomitant to impaired insulin action and decreased peripheral glucose uptake, in healthy lean males, which could not be ameliorated by RJT. Investigating changes in individual metabolic responses has provided insights into alterations in circulating adropin, irisin, and RBP4, as well as insulin sensitivity, following prolonged inactivity and high levels of sedentary time. In the subgroup with decreased insulin sensitivity after HDT bed rest, there was a significant increase in RBP4 and decreases in adropin and irisin, in addition to evidence of blunted insulin action and a marked impairment in peripheral glucose uptake. Conversely, in the subgroup with increased insulin sensitivity post-HDT bed rest, RBP4, adropin, and irisin did not change, but there was a significant improvement in liver insulin sensitivity, suggesting that the improvement in whole-body insulin sensitivity was an indirect effect of liver metabolism. We propose that RBP4, adropin, and irisin, in addition to fetuin-A, are candidate biomarkers for providing insights into whole-body and tissue-specific insulin sensitivity and tracking changes in physiological responsiveness to a gravity deprivation intervention in a lean male cohort.

## Data Availability

The raw data supporting the conclusions of this article will be made available by the authors, without undue reservation.

## Abbreviations

AUCG, area under the curve for glucose; AUCI, area under the curve for insulin; BDC, baseline data collection; BMI, body mass index; CTRL, control group; ELISA, enzyme-linked immunosorbent assay; HDL, high-density lipoprotein; HDT, head-down-tilt; JUMP, jump countermeasure group; LDL, low-density lipoprotein; OGTT, oral glucose tolerance test; RBP4, retinol binding protein-4; R, recovery; RJT, reactive jump training; SD, standard deviation; PV, plasma volume; T2DM, type 2 diabetes mellitus; $\dot{V}$ O_2peak_, peak aerobic capacity.
