# Supplementary material for: The effect of 60 days of 6° head-down-tilt bed rest on circulating adropin, irisin, retinol binding protein-4 (RBP4) and individual metabolic responses in young, healthy males
Source: Front Physiol. 2024 Sep 10;15:1435448. doi: 10.3389/fphys.2024.1435448 (PMC11420021; doi:10.3389/fphys.2024.1435448)
Supplement: Supplementary file 1 [file Table1.docx]

*Supplementary Material*

Table 1. The effect of 60 days HDT bed rest on glucose tolerance and insulin sensitivity.

| Measurement | CTRL (n = 11) | | JUMP (n = 12) | | Statistics | | |
| --- | --- | --- | --- | --- | --- | --- | --- |
|  | **Pre** | **Post** | **Pre** | **Post** | **Time** | **Group** | **T*G** |
| Glucose_0_ (mmol/L) | 5.18 ± 0.40 | 5.24 ± 0.51 | 5.32 ± 0.64 | 5.47 ± 0.59 | 0.447 | 0.311 | 0.732 |
| Glucose_30_ (mmol/L) | 8.77 ± 1.25 | 9.28 ± 0.99 | 8.62 ± 1.53 | 9.08 ± 1.22 | 0.114 | 0.691 | 0.933 |
| Glucose_60_ (mmol/L) | 6.77 ± 1.67 | 8.49 ± 1.33 | 7.90 ± 2.06 | 8.99 ± 1.87 | **<0.001** | 0.233 | 0.332 |
| Glucose_90_ (mmol/L) | 7.67 ± 1.59 | 8.11 ± 1.96 | 7.90 ± 2.52 | 8.62 ± 1.69 | 0.126 | 0.628 | 0.704 |
| Glucose_120_ (mmol/L) | 6.95 ± 1.01 | 8.51 ± 1.80 | 7.36 ± 1.82 | 7.98 ± 1.98 | **0.010** | 0.919 | 0.234 |
| AUCG_30_ (mmol/L*min) | 209.17 ± 22.30 | 217.80 ± 20.08 | 208.99 ± 27.29 | 218.30 ± 25.83 | 0.133 | 0.985 | 0.953 |
| AUCG_120_ (mmol/L*min) | 878.09 ± 128.83 | 982.98 ± 140.28 | 922.54 ± 187.30 | 1002.46 ± 154.12 | **0.006** | 0.583 | 0.681 |
| Insulin­_0_ (pmol/L) | 45.00 ± 13.09 | 51.48 ± 16.49 | 55.55 ± 14.92 | 67.22 ± 19.14 | **0.012** | **0.036** | 0.437 |
| Insulin_30_ (pmol/L) | 466.73 ± 237.48 | 539.37 ± 165.34 | 556.61 ± 289.34 | 456.43 ± 157.69 | 0.771 | 0.965 | 0.077 |
| Insulin_60_ (pmol/L) | 418.55 ± 220.00 | 490.29 ± 217.96 | 454.56 ± 220.72 | 536.05 ± 190.38 | 0.060 | 0.614 | 0.901 |
| Insulin_90_ (pmol/L) | 454.79 ± 272.03 | 500.08 ± 238.30 | 514.60 ± 228.79 | 561.16 ± 249.70 | 0.154 | 0.546 | 0.984 |
| Insulin_120_­† (pmol/L) | 347.16 ± 184.12 | 496.38 ± 321.36 | 468.39 ± 281.21 | 492.68 ± 260.41 | 0.054 | 0.587 | 0.264 |
| AUCI_30_ (pmol/L*min) | 7676.03 ± 3684.34 | 8862.74 ± 2636.68 | 9182.33 ± 4426.95 | 7856.15 ± 2567.66 | 0.922 | 0.843 | 0.089 |
| AUCI_120_ (pmol/L*min) | 46084.83 ± 20023.15 | 54110.26 ± 16896.34 | 53632.08 ± 17872.47 | 55010.45 ± 15743.61 | **0.031** | 0.558 | 0.117 |

Data are presented as mean ± SD. Metabolic characteristics were measured on BDC-5 and HDT59. Metabolic characteristics were corrected for hemoconcentration post-HDT bed rest. Significant p-values (p ≤ 0.05) are indicated in bold. A dagger (†) denotes that data was transformed for statistical analysis. Abbreviations: CTRL, control group; JUMP, jumping countermeasure group; Time, main effect of time; Group, main effect of group; T*G, time*group interaction effect; Glucose_0_, fasting glucose; Glucose_120_, glucose concentrations 120 minutes after the glucose load; AUCG, area under the curve for glucose; Insulin_0_, fasting insulin; Insulin_120_, insulin concentrations 120 minutes after the glucose load, AUCI, area under the curve for insulin.

Table 2. The effect of 60 days HDT bed rest on measures of the lipid profile and lipid ratios.

| Measurement | CTRL (n = 11) | | JUMP (n = 12) | | Statistics | | |
| --- | --- | --- | --- | --- | --- | --- | --- |
|  | **Pre** | **Post** | **Pre** | **Post** | **Time** | **Group** | **T*G** |
| NEFA (mmol/L) | 0.40 ± 0.13 | 0.41 ± 0.16 | 0.41 ± 0.14 | 0.52 ± 0.17 | 0.124 | 0.232 | 0.215 |
| TG (mmol/L) | 0.87 ± 0.26 | 1.00 ± 0.26 | 1.16 ± 0.45 | 1.21 ± 0.41 | **0.013** | 0.102 | 0.245 |
| CHOL (mmol/L) | 4.09 ± 0.72 | 4.27 ± 0.86 | 4.11 ± 0.57 | 4.06 ± 0.57 | 0.619 | 0.710 | 0.381 |
| HDL (mmol/L) | 1.16 ± 0.17 | 0.99 ± 0.19 | 1.08 ± 0.27 | 0.91 ± 0.17 | **<0.001** | 0.304 | 0.955 |
| LDL (mmol/L) | 2.76 ± 0.71 | 3.10 ± 0.71 | 2.73 ± 0.51 | 2.99 ± 0.56 | **0.004** | 0.780 | 0.647 |

Data are presented as mean ± SD. Metabolic characteristics were measured on BDC-5 and HDT59. Metabolic characteristics were corrected for hemoconcentration post-HDT bed rest. Significant p-values (p ≤ 0.05) are indicated in bold. Abbreviations: CTRL, control group; JUMP, jumping countermeasure group; Time, main effect of time; Group, main effect of group; T*G, time*group interaction effect; NEFA, non-esterified fatty acids; TG, triglycerides; CHOL, total cholesterol; HDL, high-density lipoprotein cholesterol; LDL, low-density lipoprotein cholesterol.

Table 3. The effect of 60 days HDT bed rest on estimates of insulin sensitivity and insulin resistance.

| Measurement | CTRL (n = 11) | | JUMP (n = 12) | | Statistics | | |
| --- | --- | --- | --- | --- | --- | --- | --- |
|  | **Pre** | **Post** | **Pre** | **Post** | **Time** | **Group** | **T*G** |
| Matsuda | 5.44 ± 2.12 | 4.24 ± 1.21 | 4.37 ± 1.87 | 3.52 ± 0.99 | **0.003** | 0.125 | 0.720 |
| Liver Insulin Sensitivity | 0.74 ± 0.29 | 0.64 ± 0.20 | 0.57 ± 0.18 | 0.46 ± 0.11 | **0.021** | **0.025** | 0.886 |

Data are presented as mean ± SD. Metabolic characteristics were measured on BDC-5 and HDT59. Metabolic characteristics were corrected for hemoconcentration post-HDT bed rest. Significant p-values (p ≤ 0.05) are indicated in bold. Abbreviations: CTRL, control group; JUMP, jumping countermeasure group; Time, main effect of time; Group, main effect of group; T*G, time*group interaction effect.

Table 4. The effect of 60 days HDT bed rest on glucose tolerance and insulin sensitivity when subjects were divided into two subgroups based on a decrease or an increase in insulin sensitivity post-HDT bed rest.

| Measurement | Decreased Insulin Sensitivity Subgroup  (n = 17) | | p-value | Increased Insulin Sensitivity Subgroup  (n = 6) | | p-value |
| --- | --- | --- | --- | --- | --- | --- |
|  | **Pre** | **Post** |  | **Pre** | **Post** |  |
| Glucose_0_ (mmol/L) | 5.15 ± 0.39 | 5.50 ± 0.51 | **0.011** | 5.52 ± 0.80 | 4.96 ± 0.47 | 0.058 |
| Glucose_30_ (mmol/L) | 8.56 ± 1.47 | 9.31 ± 1.09 | 0.060 | 9.05 ± 1.08 | 8.80 ± 1.14 | 0.183 |
| Glucose_60_ (mmol/L) | 7.61 ± 2.09 | 9.13 ± 1.48 | **0.001** | 6.64 ± 1.27 | 7.69 ± 1.65 | 0.126 |
| Glucose_90_ (mmol/L) | 7.88 ± 2.37 | 8.88 ± 1.62 | **0.029** | 7.53 ± 0.99 | 6.94 ± 1.57 | 0.240 |
| Glucose_120_ (mmol/L) | 7.17 ± 1.65 | 8.61 ± 1.70 | **0.005** | 7.15 ± 0.89 | 7.17 ± 2.11 | 0.978 |
| AUCG_30_ (mmol/L*min) | 205.73 ± 24.00 | 222.21 ± 21.81 | **0.023** | 218.55 ± 25.40 | 206.30 ± 22.99 | **0.039** |
| AUCG_120_ (mmol/L*min) | 906.41 ± 178.31 | 1031.41 ± 122.54 | **0.002** | 886.73 ± 102.75 | 884.73 ± 157.82 | 0.957 |
| Insulin_0_ (pmol/L) | 49.23 ± 15.91 | 62.78 ± 20.65 | **0.003** | 54.11 ± 11.35 | 50.94 ± 12.06 | 0.279 |
| Insulin_30_ (pmol/L) | 450.31 ± 244.17‡ | 470.47 ± 152.35 | 0.750 | 693.00 ± 251.45 | 568.91 ± 185.44 | **0.046** |
| Insulin_60_ (pmol/L) | 449.39 ± 223.39 | 513.30 ± 181.50 | 0.167 | 403.20 ± 209.50 | 516.63 ± 267.81 | 0.201 |
| Insulin_90_ (pmol/L) | 499.65 ± 275.99 | 569.01 ± 265.60 | 0.084 | 447.30 ± 145.26 | 426.94 ± 106.70 | 0.622 |
| Insulin_120_ (pmol/L) | 428.44 ± 279.31 | 542.63 ± 309.92 | 0.062 | 359.35 ± 68.99 | 357.95 ± 134.68 | 0.984 |
| AUCI_30_ (pmol/L*min) | 7493.21 ± 3761.01 | 7998.69 ± 2478.20 | 0.594 | 11206.63 ± 3920.85 | 9297.70 ± 2902.07 | **0.041** |
| AUCI_120_ (pmol/L*min) | 49145.69 ± 20498.88 | 55664.21 ± 16667.60 | **0.027** | 52506.88 ± 14672.98 | 51507.78 ± 14590.59 | 0.477 |

Data are presented as mean ± SD. Metabolic characteristics were measured on BDC-5 and HDT59. Metabolic characteristics were corrected for hemoconcentration post-HDT bed rest. Significant p-values (p ≤ 0.05) are indicated in bold. A double dagger (‡) represents a significant difference between subgroups at baseline. Abbreviations: Glucose_0_, fasting glucose; Glucose_120_, glucose concentrations 120 minutes after the glucose load; AUCG, area under the curve for glucose; Insulin_0_, fasting insulin; Insulin_120_, insulin concentrations 120 minutes after the glucose load, AUCI, area under the curve for insulin.

Table 5. The effect of 60 days HDT bed rest on measures of the lipid profile and lipid ratios when subjects were divided into two subgroups based on a decrease or an increase in insulin sensitivity post-HDT bed rest.

| Measurement | Decreased Insulin Sensitivity Subgroup  (n = 17) | | p-value | Increased Insulin Sensitivity Subgroup  (n = 6) | | p-value |
| --- | --- | --- | --- | --- | --- | --- |
|  | **Pre** | **Post** |  | **Pre** | **Post** |  |
| NEFA (mmol/L) | 0.42 ± 0.14 | 0.50 ± 0.19 | 0.137 | 0.35 ± 0.09 | 0.38 ± 0.07 | 0.667 |
| TG (mmol/L) | 1.02 ± 0.40 | 1.14 ± 0.38 | **0.009** | 1.03 ± 0.39 | 1.03 ± 0.30 | 0.969 |
| CHOL (mmol/L) | 4.14 ± 0.51 | 4.23 ± 0.66 | 0.519 | 4.00 ± 0.95 | 3.96 ± 0.90 | 0.887 |
| LDL (mmol/L) | 2.75 ± 0.40 | 3.09 ± 0.53 | **0.003** | 2.73 ± 1.03 | 2.92 ± 0.89 | 0.440 |
| HDL (mmol/L) | 1.13 ± 0.26 | 0.98 ± 0.19 | **0.007** | 1.09 ± 0.14 | 0.87 ± 0.10 | **0.003** |

Data are presented as mean ± SD. Metabolic characteristics were measured on BDC-5 and HDT59. Metabolic characteristics were corrected for hemoconcentration post-HDT bed rest. Significant p-values (p ≤ 0.05) are indicated in bold. Abbreviations: NEFA, non-esterified fatty acids; TG, triglycerides; CHOL, total cholesterol; HDL, high-density lipoprotein cholesterol; LDL, low-density lipoprotein cholesterol.

Table 6. The effect of 60 days HDT bed rest on estimates of insulin sensitivity and insulin resistance when subjects were divided into two subgroups based on a decrease or an increase in insulin sensitivity post-HDT bed rest.

| Measurement | Decreased Insulin Sensitivity Subgroup  (n = 17) | | p-value | Increased Insulin Sensitivity Subgroup  (n = 6) | | p-value |
| --- | --- | --- | --- | --- | --- | --- |
|  | **Pre** | **Post** |  | **Pre** | **Post** |  |
| Matsuda | 5.16 ± 2.28 | 3.64 ± 1.20 | **<0.001** | 4.09 ± 0.65 | 4.51 ± 0.59 | **0.011** |
| Liver Insulin Sensitivity | 0.70 ± 0.28‡ | 0.51 ± 0.19 | **0.001** | 0.54 ± 0.08 | 0.64 ± 0.10 | **0.012** |

Data are presented as mean ± SD. Metabolic characteristics were measured on BDC-5 and HDT59. Metabolic characteristics were corrected for hemoconcentration post-HDT bed rest. Significant p-values (p ≤ 0.05) are indicated in bold. A double dagger (‡) represents a significant difference between subgroups at baseline.
